# Long-Term Stabilization of Dengue Virus RNA at 37 °C for 14 Months Using Silk Fibroin Films

**DOI:** 10.3390/v17111452

**Published:** 2025-10-31

**Authors:** Nuo Wang, Ping He, Bohan Xu, Hongping Wei, Junping Yu

**Affiliations:** 1 State Key Laboratory of Virology and Biosafety, Wuhan Institute of Virology, Chinese Academy of Sciences, Wuhan 430071, China; wangnuo@wh.iov.cn (N.W.); xubohan22@mails.ucas.ac.cn (B.X.); 2 Chongqing Key Laboratory of Highly Pathogenic Microbes, Chongqing Disease Prevention and Public Health Research Center Construction Program, Chongqing Center for Disease Control and Prevention (Chongqing Academy of Preventive Medicine), Chongging 400707, China; peace192@163.com; 3 University of Chinese Academy of Sciences, Beijing 100049, China

**Keywords:** positive controls, RNA, dengue virus, silk fibroin film, stability

## Abstract

Diagnosis of dengue virus infections typically relies on RT-PCR-based methods, for which reliable positive controls are essential. Viral RNA is an ideal positive control, but its inherent instability poses a major challenge. Herein, we report a simple and effective method for stabilizing dengue virus RNA by immobilizing it onto silk fibroin films (RNA-SFFs). We evaluated various substrate surfaces for RNA-SFFs preparation and found that the inner surface of sealable bags is optimal for uniform film formation and easy harvesting. Screening different silk fibroin concentrations revealed that even low concentrations (2.8%) effectively preserved RNA well and kept Ct constant for up to 16 days at 25 °C, 37 °C, and even 45 °C (extreme weather for transportations). Due to its rapid film formation and ease of peeling, 7% silk fibroin was selected. Notably, the RNA-SFFs demonstrated robust resistance to UV irradiation, with no significant Ct value changes after 4 h of exposure. Long-term stability testing at −20 °C, 25 °C, and 37 °C showed that dengue serotype 1–4 RNA-SFFs remained stable for the entire duration of the study—up to 56 weeks (approximately 14 months)—at all tested temperatures. These results demonstrate that RNA-SFFs are highly stable, portable, and practical as positive controls for dengue diagnostics, with strong potential for use in on-site and resource-limited settings.

## 1. Introduction

Dengue, an arboviral disease caused by dengue viruses and transmitted by *Aedes* mosquitoes, can lead to mild or severe sickness. The symptoms include fever, aches (such as eye pain, typically behind the eyes, muscle, joint, or bone pain), nausea or rashes. Approximately 5% of individuals who develop dengue will progress to severe dengue, which may result in shock, internal bleeding, and even death [1,2]. The dengue virus comprises four distinct serotypes (DENV-1 to DENV-4), contributing to its complex epidemiology. In 2024, the World Health Organization’s (WHO) reported a record 14.4 million dengue cases globally (https://worldhealthorg.shinyapps.io/dengue_global/, accessed on 15 October 2025), which is more than double the 7 million cases recorded in 2023 [3]. The real situation might be more serious. One modeling estimate indicates that dengue fever affects more than 400 million people annually worldwide and causes around 22,000 deaths across over 100 countries. Given the lack of specific antiviral treatment, early diagnosis continues to play a critical role in enabling timely treatment and minimizing the risk of severe dengue [2,4].

Reverse transcription-polymerase chain reaction (RT-PCR) remains the benchmark method for the early and accurate detection of dengue virus infection [5,6]. To ensure the reliability and validity of RT-PCR, the inclusion of positive controls or reference materials in each run is essential. Genomic RNA has become a widely adopted reference standard in molecular diagnostics and assay calibration due to its full-length genomic sequence, ease of production, and suitability for scalable manufacturing [7,8]. However, RNA molecules are inherently unstable and highly susceptible to enzymatic (e.g., ribonuclease-mediated) and chemical degradation (e.g., hydrolysis, oxidation), necessitating immediate stabilization via storage at ultra-low temperatures or under desiccated conditions [7,9,10,11]. This requirement presents significant challenges for resource-limited settings.

To circumvent these constraints, various ambient-temperature stabilization strategies have been investigated. Lyophilization (freeze-drying) [12], encapsulation within metallic capsules [10,11], or entrapment in silica-based microparticles [13] offer enhanced stability but are often prohibitively expensive, labor-intensive, or ill-suited for deployment in resource-limited or field settings.

Silk fibroin is a natural protein polymer derived from the cocoons of the domesticated silkworm *Bombyx mori*. In its native state, silk fibroin adopts a semicrystalline architecture comprising approximately 65% crystalline domains and 35% amorphous regions, which synergistically contribute to the mechanical resilience and structural robustness of the cocoons. Silk fibroin exhibits high solubility in water and can be processed into a regenerated material with a water-stable structure, featuring a predominant β-sheet crystalline conformation. This conformation endows the protein-based matrix with exceptional thermal stability, mechanical strength under tension and resistance to chemical degradation [14,15]. Owing to these unique physicochemical properties—particularly its biocompatibility and ability to stabilize labile biomolecules—silk fibroin has emerged as a highly promising matrix for the encapsulation and preservation of thermosensitive biological agents, including whole blood, DNA, and RNA [14,16,17,18,19,20,21].

In this study, silk fibroin is employed to form films incorporating dengue viral RNA, serving as reference materials to support the development and evaluation of RT-PCR-based dengue virus diagnostic assays. The resulting RNA-silk fibroin films (RNA-SFFs) effectively stabilize dengue viral RNA, with no significant change in RT-qPCR Ct values observed after storage for at least 14 months at 37 °C (longer durations have not yet been evaluated). RNA-SFFs prepared from various concentrations of silk fibroin maintain RNA stability at 25 °C, 37 °C and 45 °C for 16 days. Additionally, the RNA-SFFs exhibit resistance to UV radiation, as evidenced by stable Ct values following 4 h of exposure to UV light at an intensity of over 90 μW/cm^2^. These results suggest minimal RNA degradation under thermal and environmental stress. Therefore, the developed RNA-SFFs represent a promising cold-chain-free alternative for the long-term storage and transport of reference materials, with potential utility in resource-limited and field-deployable diagnostic settings.

## 2. Materials and Methods

### 2.1. Materials

Dengue virus serotypes 1 through 4 were obtained from clinical specimens derived from infected individuals and propagated in *Aedes albopictus* C6/36 cells (IVCAS9.087 in National Virus Resource Center, Wuhan Institute of Virology, CAS, Wuhan, China). C6/36 cells were cultured in T75 flasks using MEM supplemented with 10% fetal bovine serum (FBS) and 1% penicillin-streptomycin. When cells reached 70–80% confluence, they were infected with DENV-1 to DENV-4 at a multiplicity of infection (MOI) ranging from 0.01 to 0.1. The infected cultures were incubated at 37 °C in a 5% CO_2_ humidified incubator until pronounced cytopathic effect (CPE) was observed (3–4 days). Viral supernatants were then collected for RNA extraction. Although the initial infectious virus titer was not determined by plaque assay or TCID_50_, the viral load in the supernatant was assessed by RT-qPCR, yielding Ct values between 10 and 15, indicating high levels of viral RNA. Viral nucleic acids were isolated utilizing the QIAamp^®^ Viral RNA Mini Kit (Qiagen, Hilden, Germany), following the manufacturer’s protocol. The HiScript^®^ II One Step qRT-PCR Probe Kit (Vazyme Biotech Co., Ltd., Nanjing, China) was used for RT-qPCR. Cocoons were purchased from Crystalgen Ningbo Biotech Ltd. (Ningbo, China). Lithium bromide and sodium carbonate were bought from Shanghai Aladdin Biochemical Technology Co., Ltd. (Shanghai, China) and Sinopharm Chemical Reagent Co., Ltd. (Shanghai, China). All primers and fluorescently labeled probes were commercially synthesized by Sangon Biotech Co., Ltd. (Shanghai, China). Nuclease-free water, essential for molecular assays to prevent degradation of nucleic acids, was sourced from Source Leaf Biotech Co., Ltd. (Shanghai, China). The primer/probe sets for the four dengue virus serotypes 1 through 4 are listed in Table 1. Ultrapure water with a resistivity of 18.2 MΩ·cm was employed in all experiments.

### 2.2. Preparation and Quantification of Silk Fibroin Solution from Silk Cocoons

Silk fibroin solution was prepared following the established protocol described by Rockwood D. et al. [16], with minor modifications. Approximately 2.5 g of *Bombyx mori* silk cocoons were manually cut into fragments of nail-clipping size using sterilized scissors. To facilitate degumming, 2.12 g of sodium carbonate was dissolved in 1 L of ultrapure water, which was heated to boiling to dissolve. The cocoon pieces were introduced into the boiling sodium carbonate solution and stirred vigorously at 110–200 rpm for 50 min to remove sericin. Following cooling, the degummed silk fibers were retrieved, transferred to 1 L of fresh ultrapure water, and subjected to magnetic stirring for 20 min to remove residual salts. This washing step was repeated three times with complete water replacement. The purified fibers were then dried overnight in a fume hood. The dried silk fibroin was weighed (about 1.70 g). For dissolution, 8.1 g of lithium bromide (LiBr) was dissolved in 10 mL of ultrapure water. The dried fibers were solubilized in the LiBr solution at a ratio of 1:4 (for example, 1.7 g silk fibers to 6.8 mL LiBr solution), followed by incubation at 60 °C for 4 h to ensure complete dissolution of the protein matrix. The resulting solution was dialyzed against 1 L of ultrapure water at 4 °C for 72 h, with the dialysate replaced three times daily to eliminate residual LiBr. After dialysis, the aqueous silk fibroin solution was centrifuged at 9000× *g* and 4 °C for 20 min to remove insoluble aggregates. The supernatant was collected and subjected to a second centrifugation under identical conditions to ensure maximal clarity. The purified silk fibroin solution was stored at 4 °C for up to one month and used in subsequent experiments as required. The concentration of the prepared silk fibroin solution was determined as follows. A clean Petri dish lid was pre-weighed (mass recorded as m_1_ in grams). Exactly 500 μL of the silk fibroin solution was pipetted onto the lid and dried at 60 °C for 1 h to constant weight. The lid with dried fibroin was weighed (mass recorded as m_2_ in grams, and the mass difference in mass (m_2_ − m_1_). The concentration of the prepared silk fibroin solution is calculated as:

$$c_{\mathrm{silk}\;\mathrm{fibroin}}\;=\;\frac{m_{2}-m_{1}}{0.5}$$

The unit of the protein concentration is in % *w*/*v*. For example, if the mass difference is 0.035 g, then the concentration is 0.035 divided by 0.5, which is 7.0% *w*/*v*.

### 2.3. Evaluation of Film Formation Efficiency of the Silk Fibroin on Various Substrate Surfaces

The prepared silk fibroin solution in Section 2.2 was quantified and adjusted to a final concentration of 7.0% *w*/*v*. To investigate the influence of substrate properties on film formation and ease of harvest, 10 μL of RNase-free water was mixed with 10 μL of the 7.0% *w*/*v* silk fibroin solution. The resulting blend was cast onto various surfaces to form circular films with a diameter of approximately 1 cm, including a Petri dish (plastic), a Petri dish (glass), aluminum foil, the inner surface of an incised new sealable plastic bag, and the interior of microcentrifuge tubes. Samples were air-dried under ambient conditions to form thin solid films. The time required for complete film formation and the ease of film detachment were assessed for each substrate.

### 2.4. RT-qPCR System and Protocol

The RT-qPCR assay was carried out in a total reaction volume of 20 μL, comprising 5 μL of template RNA, 10 μL of 2× one-step reaction mix, 1 μL of enzyme mix, 0.4 μL each of 10 μM forward and reverse primers, 0.4 μL of 10 μM fluorescent probe, and nuclease-free water to achieve the final volume. The RT-qPCR reactions were run in duplicate. Amplification and fluorescence detection were performed using the CFX96 Real-Time PCR Detection System (Bio-Rad, Hercules, CA, USA) or MA-6000 (Yarui Biotech, Suzhou, China) under the following thermal cycling conditions: an initial reverse transcription phase at 50 °C for 15 min, followed by initial denaturation at 95 °C for 30 s. This was succeeded by 45 consecutive cycles of amplification, each consisting of a denaturation step at 95 °C for 10 s and an annealing/extension step at 60 °C for 30 s, with fluorescence acquisition occurring during the latter phase of each cycle. The Ct thresholds for all qPCR runs were set automatically by the instrument’s software (Bio-Rad CFX Manager version 3.1, Hercules, CA, USA) or MA-6000 (Yarui Biotech, Suzhou, China).

### 2.5. The Effect of Silk Fibroin Films (SFFs) on Ct Values of DENV-1

Evaluation of the effect of silk fibroin on Ct values of DENV-1 RNA through RT-qPCR: To assess potential matrix-induced effects of silk fibroin on RT-qPCR quantification, a dilution series of silk fibroin solutions was prepared from 7.0% *w*/*v* using a two-fold serial dilution method, yielding concentrations of 7.0%, 3.5%, 1.75%, 0.88%, and 0.44% *w*/*v*. Here, a wide range of the silk fibroin concentrations were used to identify the threshold at which silk fibroin might begin to interfere with RT-qPCR. For each concentration, 10 μL of DENV-1 RNA was mixed with 10 μL of the respective silk fibroin solution, resulting in a 1:1 (*v*/*v*) RNA-silk matrix. The mixtures were then directly subjected to RT-qPCR analysis without prior processing.

**Figure 1:**
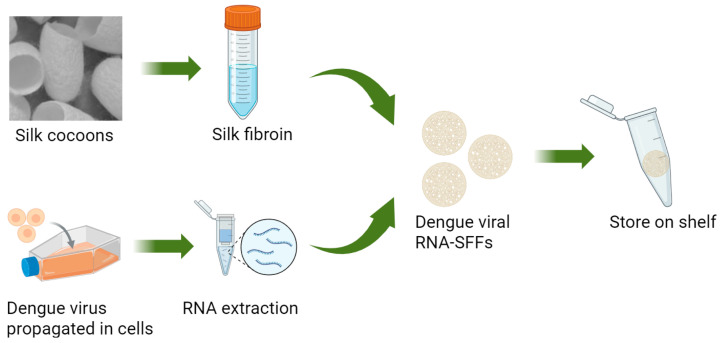
Experimental strategy for the preparation of dengue virus RNA–SFFs. Created in BioRender. Yu, J. (2025) https://app.biorender.com/illustrations/68d0ad9ed6333e7d2837d168?slideId=8df1f916-9599-42dd-8075-1a73ac3418d5 accessed on 29 October 2025.

Assessment of DENV-1 RNA stability in the form of RNA-silk fibroin films (RNA-SFFs) prepared from silk fibroin solutions of varying concentrations: Silk fibroin solutions at concentrations of 7.0%, 5.6%, 4.2% and 2.8% *w*/*v* were prepared by serial dilution of the stock solution with nuclease-free water (Source Leaf Biotech Co., Ltd., Shanghai, China), which are different from the concentrations used above due to that very low concentrations may compromise film integrity. The mixtures of 10 μL of DENV-1 viral RNA and 10 μL of each silk fibroin formulation were spread onto the inner surfaces of sealed polyethylene bags and air-dried under ambient conditions for 30 min to 1 h to yield thin, solid composite films. The resulting RNA-SFFs were transferred into 1.5 mL microcentrifuge tubes and stored under controlled thermal conditions at 25 °C, 37 °C and 45 °C. The preparation process of dengue virus RNA–silk fibroin films (SFFs) is illustrated in Figure 1. At days 1, 3, 6, 9, 13 and 16, individual samples were retrieved, and each film was rehydrated by adding 500 μL of RNase-free water. Samples were vortexed vigorously to ensure complete dissolution and homogeneous resuspension of RNA. The recovered RNA was then immediately subjected to RT-qPCR, and the cycle threshold (Ct) values were recorded.

### 2.6. Assessment of DENV-1 RNA Stability in the Form of RNA-SFFs

To evaluate the protective effect of RNA in the form of RNA-SFFs against UV-induced degradation, samples were exposed to UV radiation at an irradiance intensity exceeding 90 μW/cm^2^ for durations of 1 h, 2 h, and 4 h. The irradiance level was chosen to reflect typical conditions used for surface decontamination in biological safety cabinets and clean benches. DENV-1 RNA-SFFs were prepared following the protocol outlined in Section 2.5, and naked DENV-1 RNA in solution served as the control.

### 2.7. Long-Term Storage of the Prepared DENV-1–4 RNA-SFFs at −20 °C, 25 °C and 37 °C

Dengue virus serotypes 1–4 RNA were individually encapsulated into SFFs using the protocol described above. The prepared RNA-SFFs were stored at −20 °C, 25 °C, and 37 °C to assess long-term RNA stability under varying thermal conditions. No special precautions against light exposure were taken during storage, and temperature fluctuations were kept to a minimum to ensure consistent experimental conditions. At 1, 2, 3, 4, 6, 8, 12, 24, 48 and 56 weeks, individual films were removed from storage and dissolved in RNase-free water according to the rehydration procedure mentioned above. The dissolved RNA was then subjected to serotype-specific RT-qPCR using corresponding primer-probe sets as listed in Table 1. Ct values were recorded at each time point to monitor Dengue RNA stability.

### 2.8. Data Statistics

Data are presented as the mean ± standard deviation (SD) derived from three independent replicates. Statistical significance between experimental groups at different temperature was assessed by one-way ANOVA (and nonparametric or mixed) with Dunn’s multiple comparisons test using GraphPad Prism 9.0.0 (GraphPad Software Inc., San Diego, CA, USA). A probability value of *p* < 0.05 was designated as statistically significant.

## 3. Results

### 3.1. Selection of Appropriate Substrate Surfaces to Form SFFs

Initially, the film formation efficiency of silk fibroin was evaluated across various substrate surfaces. The results are summarized in Table 2. To optimize the preparation process of SFFs, an ideal substrate was sought that would enable the formation of intact, easily detachable films with a short air-drying time. As shown in Table 2, the inner surface of an incised new sealable plastic bag exhibited favorable characteristics: rapid air-drying, formation of structurally uniform films, and facile delamination without fragmentation. Given these advantages, this substrate was adopted for the preparation of RNA-loaded silk fibroin films (RNA-SFFs), where the RNA is embedded within the film matrix during casting and dried into a stable, peelable sheet for subsequent rehydration and use in RT-qPCR.

### 3.2. Assessment of the Effect of Silk Fibroin at Varying Concentrations on DENV-1 Genomic RNA Detection

**Figure 2:**
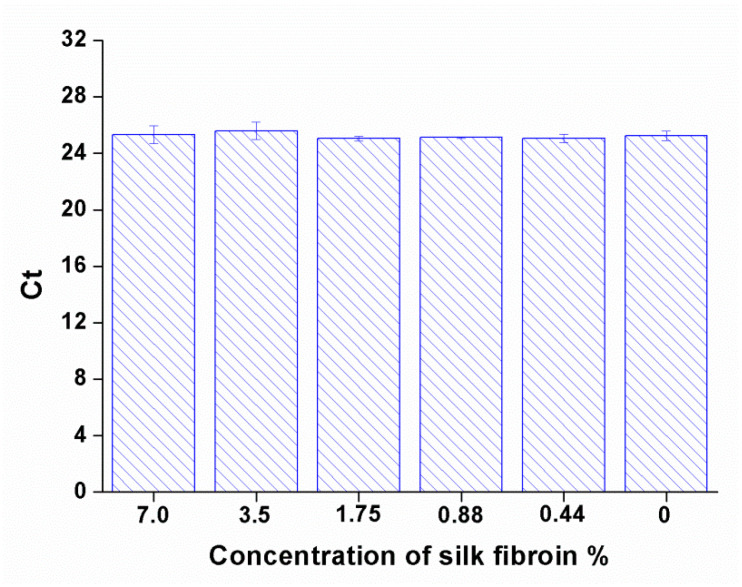
Impact of silk fibroin concentration on the RT-qPCR Ct values of DENV-1 RNA. Data are presented as the mean Ct value from three independent experiments, with error bars indicating the standard deviation (SD). Each concentration of silk fibroin (7.0%, 3.5%, 1.75%, 0.88%, and 0.44% *w*/*v*) was tested in a 1:1 (*v*/*v*) mixture with DENV-1 RNA. The control group is DENV-1 RNA with 0% silk fibroin. No significant variation in Ct values was observed across the concentration range. Statistical significance was determined by ANOVA vs. 0% silk fibroin (*p* values and z values, which reflect the magnitude of the group difference, are listed in the Supplementary Materials). No significance was observed for all the tested silk fibroin concentrations (*p* > 0.05).

We first evaluated the effect of silk fibroin on Ct values of DENV-1 viral RNA by RT-qPCR. The results are shown in Figure 2. The figure displayed no significant differences between DENV-1 viral RNA alone (the control group is DENV-1 RNA with 0% silk fibroin) and samples containing silk fibroin at various concentrations of the silk fibroin, even at 7% *w*/*v* (the corresponding final concentration of silk fibroin in the RNA samples was 3.5%). This indicates that silk fibroin had no obvious effect on the Ct value of DENV-1 viral RNA.

### 3.3. Evaluation of the Stability of DENV-1 RNA in SFFs Prepared with Silk Fibroin at Varying Concentrations

**Figure 3:**
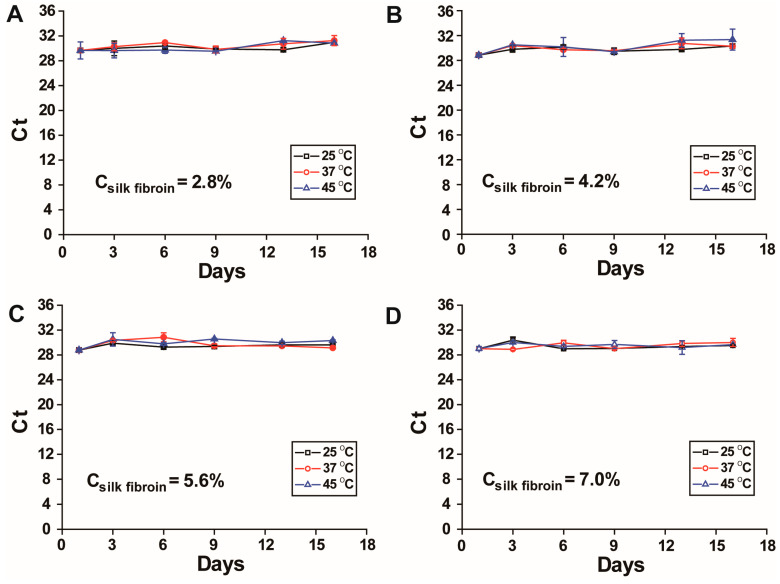
Stability of DENV-1 RNA in RNA-SFFs prepared with silk fibroin at varying concentrations. (**A**). 2.8% *w*/*v*; (**B**). 4.2% *w*/*v*; (**C**). 5.6% *w*/*v*; (**D**). 7.0% *w*/*v*. Each panel shows RT-qPCR Ct values of RNA in SFFs over time at 25 °C, 37 °C and 45 °C, indicating minimal degradation of RNA across all concentrations. The highest consistency was observed at 7.0% *w*/*v*. All data are presented as mean ± SD of three independent experiments.

DENV-1 RNA was mixed with silk fibroin solutions of four silk fibroin concentrations (7.0%, 5.6%, 4.2%, and 2.8% *w*/*v*) and dried to form RNA-silk fibroin films (RNA-SFFs) to evaluate RNA stability in the SFF format under different storage conditions at 25 °C, 37 °C and 45 °C. The results are presented in Figure 3. The data, along with statistical *p* values and z values, are provided in the Supplementary Materials. A *p* value > 0.05 indicates no significant difference, while the z value reflects the magnitude of intergroup differences. The results indicated that at all tested concentrations, RT-qPCR Ct values remained stable throughout the 16-day storage period, even under the most thermally stressful condition (45 °C), indicating that silk fibroin effectively preserves RNA well in the SFF format for at least 16 days at elevated temperatures. Notably, RNA-SFFs prepared with 7.0% *w*/*v* silk fibroin exhibited the narrowest confidence intervals and the most consistent Ct values across all time points and temperatures, as evidenced by the smallest error bars in Figure 3D (with the smallest fluctuations for all the temperatures with 7.0% *w*/*v* silk fibroin (7.0%: 29.4 ± 0.45; 5.6%: 29.7 ± 0.64; 4.2%: 30.0 ± 0.75; 2.8%: 30.2 ± 0.61). This enhanced reproducibility is likely attributable to the formation of denser, more cohesive films at the higher silk fibroin concentration, which facilitated complete and consistent delamination from the substrate surface, thereby improving sample recovery uniformity and experimental repeatability. Based on these results, a silk fibroin concentration of 7.0% *w*/*v* was selected for subsequent experiments to ensure optimal film quality and assay consistency.

### 3.4. UV Resistance of DENV-1 RNA-SFFs

**Figure 4:**
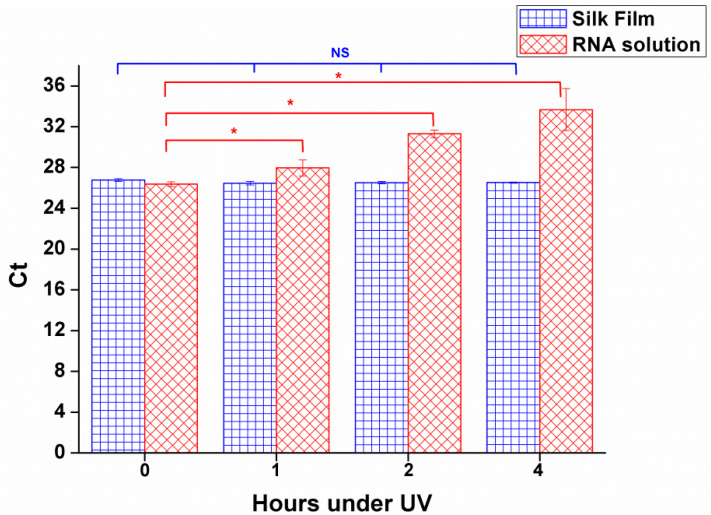
Stability of DENV-1 in RNA-SFFs and free RNA under UV irradiation at increasing exposure durations (1 h, 2 h and 4 h). Each data point represents the mean Ct value from three independent experiments, and error bars indicate SD. Statistical significance was determined by ANOVA vs. 0 h of UV irradiation. *p* and z values are displayed in the Supplementary Materials. * *p* < 0.05 for free RNA (indicating degradation); NS for RNA-SFFs (no significant change), showing effective stabilization.

DENV-1 RNA-SFFs, prepared according to the aforementioned protocol, were exposed to UV radiation at an irradiance intensity exceeding 90 μW/cm^2^, which was selected based on the typical irradiance level used for surface decontamination in biological safety cabinets and clean benches, for 1 h, 2 h and 4 h to evaluate the protective capacity of DENV-1 RNA-SFFs against UV-induced RNA degradation. In contrast to the RNA-SFFs, naked DENV-1 RNA in solution exhibited a time-dependent increase in RT-qPCR Ct values with prolonged UV exposure (Figure 4), indicating progressive RNA fragmentation and reduced amplifiability. By contrast, RNA within the silk fibroin films maintained stable Ct values across all time points, with no statistically significant differences observed after 1, 2, or 4 h of irradiation (*p* > 0.05). These results demonstrate that the silk fibroin films effectively shields RNA from UV-induced photodegradation. The RNA molecules are preserved well within the film, likely due to the physical barrier and radical-scavenging properties of the silk protein network. Thus, silk fibroin confers robust protection against environmental UV stress, highlighting its potential as a stabilizing biomaterial for RNA preservation under challenging conditions.

### 3.5. Long-Term Stability of Dengue Serotypes 1–4 RNA in RNA-SFFs Under Different Temperature Conditions

**Figure 5:**
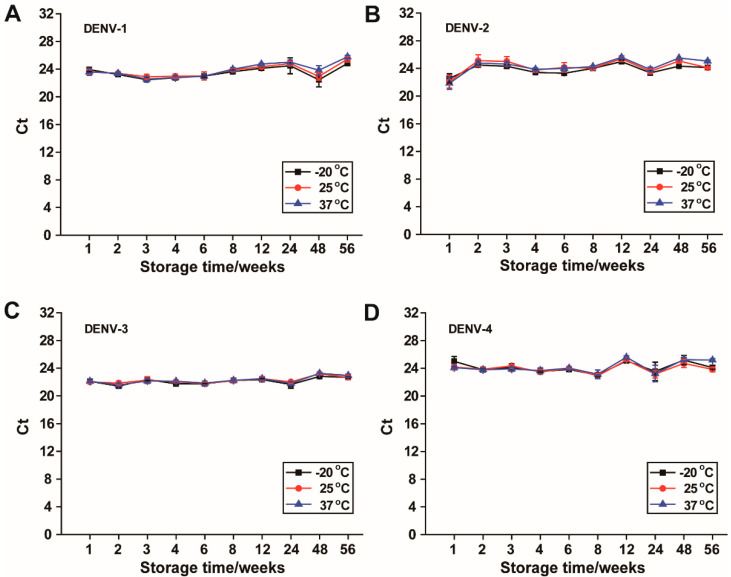
Long-term stability of the DENV-1–4 RNA in RNA-SFFs stored at −20 °C, 25 °C, 37 °C for 56 weeks (approximately 14 months). (**A**). DENV-1; (**B**). DENV-2; (**C**). DENV-3; (**D**). DENV-4. Data shown are means ± SD.

To evaluate the long-term stability of dengue viral RNA in RNA-SFFs, DENV-1–4 RNA-SFFs were stored under three distinct temperature conditions: −20 °C, 25 °C and 37 °C, for an extended period of 56 weeks (approximately 14 months). All data, including statistical *p* and z values, are available in the Supplementary Materials. Plots of Ct values vs. weeks are presented in Figure 5. The mean Ct values for the first six time points in Figure 5 are from three independent experiments, while the data for the last four time points are from two independent experiments. As shown, DENV-2 RNA (Figure 5B) exhibited a lower Ct value at the one-week time point compared to subsequent measurements. This may reflect an initial measurement variability or a transient shift in Ct values between week one and week two, after which the values stabilized and remained consistent for the remainder of the study. No significant differences were observed in Ct values between any testing time point and the reference time point (week one, or week two for DENV-2); *p* > 0.05, see Supplementary Materials (Data for Figure 5). Notably, all four dengue serotypes demonstrated excellent stability over the 56-week duration, with minimal fluctuation in Ct values across all storage temperatures. Importantly, statistical analysis revealed no significant differences in Ct values between samples stored at −20 °C and those maintained at 25 °C or 37 °C at any tested time points (*p* > 0.05). The *p* values are listed in the Supplementary Materials (Data for Figure 5). This indicates that RNA-SFFs effectively preserve RNA even under elevated, non-frozen conditions. Collectively, these findings demonstrate that RNA-SFFs provide robust protection for multivalent dengue viral RNA over prolonged periods, maintaining molecular stability for up to 56 weeks without the need for cold-chain storage. These results highlight the potential of SFFs as a versatile and reliable platform for ambient-temperature biostabilization of RNA-based diagnostics.

## 4. Discussion

Positive controls play a pivotal role in RT-PCR-based assays for the diagnosis of viral infections. Dengue viral genomic RNA serves as a critical positive control with advantages of containing all targets of dengue viruses to validate all RT-PCR-based diagnostic kits. However, RNA is inherently labile and highly susceptible to degradation by RNases, chemicals and environmental stressors such as heat and UV radiation. The storage and transport of RNA-based controls depend on cold-chain infrastructure, which limits the practical utility in resource-limited or field-deployable diagnostic settings.

In this study, we demonstrate that dengue RNA-SFFs can effectively stabilize dengue genomic RNA under challenging conditions. Specifically, RNA in SFFs remains stable for up to 14 months at 37 °C, up to 16 days at 45 °C, and for a minimum of 4 h under intense UV irradiation (>90 μW/cm^2^). These results highlight the robust protective capacity of silk fibroin matrices against both thermal and photodegradation.

Notably, we found that silk fibroin, even at a final concentration of 3.5% *w*/*v* (derived from the original 7.0% *w*/*v* solution shown in Figure 2), did not significantly affect RT-qPCR Ct values. This observation contrasts with a previous report [21], in which silk concentrations exceeding 1% *w*/*v* were shown to interfere with Ct values, although this interference was mitigated by RNA purification. The discrepancy may stem from differences in experimental conditions, including variations in silk fibroin preparation methods, PCR master mix composition, or RNA type. Importantly, in our system, RNA can be directly detected from the dissolved films without prior extraction, enabling a simpler and more user-friendly workflow. Consistent with our findings, RNA in SFFs remained stable at elevated temperatures, including up to 45 °C, further supporting the potential for using RNA-SFFs-based reference materials in extreme or resource-limited environments.

To the best of our knowledge, stability for 14 months at 37 °C represents the longest reported storage duration for viral RNA reference materials under non-refrigerated conditions to date. A recent study [10] demonstrated that RNA encapsulated in dehydrated form within metallic capsules remained stable for up to 3 years at room temperature, as assessed by RT-qPCR. However, that method relies on proprietary technology developed by a commercial entity. Due to intellectual property restrictions and cost considerations, this approach is not readily adaptable for use in independently developed diagnostic assays or in-house testing systems. In contrast our method demonstrated consistent RNA protection across all four dengue virus serotypes, indicating that stability is maintained regardless of genomic sequence variation. This pan-serotypic efficacy underscores the versatility and generalizability of the silk fibroin matrix, making it particularly valuable for multivalent diagnostic kits where balanced detection sensitivity across serotypes is essential.

Several limitations of this study should be acknowledged. First, while this work provides proof-of-concept, the development of RNA-SFFs-based materials into standardized reference materials will require precise quantification of dengue viral RNA copy number in the films, followed by multi-laboratory validation to ensure reproducibility and comparability across testing sites. Second, although the materials exhibited excellent long-term stability, the upper limits of stability—particularly under higher temperatures and more extreme environmental conditions—have not yet been fully defined. Further studies using accelerated aging protocols (e.g., elevated temperatures, variable humidity) are needed to establish shelf life and define optimal storage boundaries. As well, the cost-effectiveness and scalability of large-scale production, and the effects of different batches of silk fibroin preparations, and stability tests by different persons, require further investigation. In addition, the evaluation of RNA stability across a broad dynamic range, particularly at low-copy inputs (e.g., Ct > 30) that are representative of weak-positive or near-limit-of-detection diagnostic controls should be studies further. Moreover, the molecular mechanisms underlying the exceptional thermal stability of silk fibroin-RNA films, especially at temperatures up to 45 °C, remain unclear. A deeper understanding of the interactions between silk fibroin and nucleic acids, as well as the role of water exclusion dynamics during film formation could guide the rational design of more robust biomaterials. Such insights may not only improve RNA preservation but also expand the application of silk fibroin in stabilizing other labile biologics such as vaccines and biopharmaceuticals, thereby contributing to global health.

## 5. Conclusions

Genomic RNA-based positive controls are essential for current dengue virus diagnosis but are inherently labile and require stringent cold-chain storage, limiting their use in tropical and resource-limited settings. To overcome this limitation, we developed a silk fibroin film (SFF)-based platform for the stabilization of full-length dengue viral RNA. Our results show that RNA in RNA-SFFs exhibited no significant increase in RT-qPCR Ct values across all four serotypes (DENV-1–4) after more than 14 months of storage at 37 °C. Furthermore, the RNA-SFFs exhibit robust resistance to both UV irradiation and elevated temperature (45 °C for at least 16 days). These findings establish silk fibroin as a highly effective matrix for ambient-temperature preservation of viral RNA. The RNA-SFFs platform offers a promising strategy for developing ready-to-use, cold-chain-free positive controls, thereby enhancing the reliability and accessibility of molecular diagnostics in dengue-endemic regions.

## Figures and Tables

**Table 1:** Primers and probes targeting the four dengue virus serotypes for RT-qPCR.

Primer or Probe	Sequence (5′-3′)
DENV-1-F	TGTGCATTGAAGCTAAAATATCA
DENV-1-R	CGTCTTGTTCTTCCACCA
DENV-1-P	FAM-ACCACCACCGACTCAAGATGTCCAA-BHQ1
DENV-2-F	CGAGAAATACGCCTTTCAATA
DENV-2-R	CAGCATTCCAAGTGAGAATC
DENV-2-P	FAM-AACCGCGTGTCAACTGTGCAAC-BHQ1
DENV-3-F	CAACCAACGGAAGAAGAC
DENV-3-R	CGCCAACTGTGATCCAGT
DENV-3-P	FAM-AAACCGTCTATCAATATGCTGAAACGC-BHQ1
DENV-4-F	GGTTGGTGAAGAGATTCTCA
DENV-4-R	GTGGGATGGAAAGGACTC
DENV-4-P	FAM-AGCACCATCCGTAAGGGTCCT-BHQ1

**Table 2:** Evaluation of the film formation efficiency of silk fibroin on various substrate surfaces.

Substrate	Air-Drying Time/min	Film Morphology	Peelability
Petri dish (polystyrene)	60	Brittle and prone to cracking	Difficult to peel intact; required multiple attempts with residual adhesion
Petri dish (glass)	40	Powder-like morphology, no continuous film structure formed	Not peelable; only removable via scraping
Aluminum foil	40	Adhered strongly to the surface; no visible intact film	Unable to be peeled off without damaging or fragmenting
Sealable plastic bag	40	Uniform, flexible and structurally intact film	Easily removable without fragmentation
Centrifuge tube	>60	Mixture remained liquid after more than one hour, no drying or film formation observed	Not applicable

## Data Availability

All data generated in this study are included in the manuscript and its Supplementary Materials.

## Supplementary Materials

The following supporting information can be downloaded at: https://www.mdpi.com/article/10.3390/v17111452/s1, Table S1: Amplification efficiencies and limit of detection of the primer/probe sets; Data for Figure 2: Ct values and statistical P and z values for effect of silk fibroin at varying concentrations; Data for Figure 3: Ct values and statistical P and z values for different concentrations of fibroin; Data for Figure 4: Ct values and statistical P and z values for effect of UV; Data for Figure 5: Ct values and statistical P and z values for long-term effect.
